# The importance of censoring in competing risks analysis of the subdistribution hazard

**DOI:** 10.1186/s12874-017-0327-3

**Published:** 2017-04-04

**Authors:** Mark W. Donoghoe, Val Gebski

**Affiliations:** University of Sydney, NHMRC Clinical Trials Centre, Sydney, 2006 NSW Australia

**Keywords:** Censoring, Competing risks, Proportional subdistribution hazards model

## Abstract

**Background:**

The analysis of time-to-event data can be complicated by competing risks, which are events that alter the probability of, or completely preclude the occurrence of an event of interest. This is distinct from censoring, which merely prevents us from observing the time at which the event of interest occurs. However, the censoring distribution plays a vital role in the proportional subdistribution hazards model, a commonly used method for regression analysis of time-to-event data in the presence of competing risks.

**Methods:**

We present the equations that underlie the proportional subdistribution hazards model to highlight the way in which the censoring distribution is included in its estimation via risk set weights. By simulating competing risk data under a proportional subdistribution hazards model with different patterns of censoring, we examine the properties of the estimates from such a model when the censoring distribution is misspecified. We use an example from stem cell transplantation in multiple myeloma to illustrate the issue in real data.

**Results:**

Models that correctly specified the censoring distribution performed better than those that did not, giving lower bias and variance in the estimate of the subdistribution hazard ratio. In particular, when the covariate of interest does not affect the censoring distribution but is used in calculating risk set weights, estimates from the model based on these weights may not reflect the correct likelihood structure and therefore may have suboptimal performance.

**Conclusions:**

The estimation of the censoring distribution can affect the accuracy and conclusions of a competing risks analysis, so it is important that this issue is considered carefully when analysing time-to-event data in the presence of competing risks.

**Electronic supplementary material:**

The online version of this article (doi:10.1186/s12874-017-0327-3) contains supplementary material, which is available to authorized users.

## Background

Competing risks are commonly observed in time-to-event data, and there have recently been major methodological advances in regression analysis of such data. In this paper, we focus on one particular model for competing risks data: proportional subdistribution hazards regression. Censoring plays an important role in the estimation of such a model, and can thus have an impact on the subsequent results and conclusions. This issue is often not given sufficient attention in texts that introduce and discuss these methods (e.g. [1–3]), and as a result is not usually considered when the proportional subdistribution hazards model is implemented.

### Definitions

One of the key concepts in competing risks analysis is the distinction between competing risk events and censoring. We are interested in the duration *T* between the time origin and the occurrence of an event. Censoring is the process which prevents us from fully observing this *T*. In particular, right-censoring occurs when we know that an event has not occurred prior to some time *C*, but we can no longer follow the individual to measure *T* exactly, and hence only observe *Z*= min(*T*,*C*).

Time-to-event analysis methods allow us to use the information that, for censored individuals, *T*>*C*, but often rely on the crucial assumption that the censoring process is non-informative. Essentially, this means that knowledge of the censoring time for an individual gives us no additional information on their future risk of an event. This condition can be equivalently expressed in terms of the hazard of censoring, meaning that the instantaneous probability of being censored does not depend on the unknown future event time. Robins and Finkelstein [4] have discussed the conditions under which the assumption of non-informative censoring holds when there is a set of covariates that affects the risk of events and/or censoring.

Competing risks, conversely, are typically informative. As defined by Gooley et al. [5], a competing event is one which precludes the event of interest from occurring, or fundamentally changes its probability. If competing events are treated as censored observations, standard survival analysis methods can be used to estimate the effect of covariates on cause-specific hazards, which has some utility in helping to understand disease aetiology [6]. However, if there is dependence between the competing events, the cause-specific hazard cannot be interpreted as the marginal hazard and covariate effects do not directly translate onto the cumulative scale. One of the difficulties associated with competing events—and in fact, censoring in general—is that, without making restrictive assumptions about the exact nature of the dependency between the different event types, it is impossible to distinguish between dependent and independent event processes [7].

An alternative approach is to focus on analysing both the time *T* until the first event and the type of that event, denoted by *δ*. Without loss of generality, we will use *δ*=1 to denote the event of interest and *δ*=2 for any other event type.

In the following sections, we use the ‘traditional’ time-to-event notation rather than counting process notation for the sake of readability for readers without a rigorous statistical background. We also assume that the data contain no tied event times, because methods for handling ties can differ slightly between software packages, and discussion of these issues is beyond the scope of this paper.

### The subdistribution function

One quantity of interest in a competing risks analysis is the cumulative incidence function, or subdistribution: *F*(*t*)=Pr(*T*≤*t*,*δ*=1), which is the probability of experiencing the event of interest as the first event prior to time *t*. It is called the subdistribution because, if Pr(*δ*≠1)>0, ${\lim }_{t \rightarrow \infty } F(t) < 1$, and may be viewed as the distribution function of an improper random variable *τ* that takes value *T* if *δ*=1 or value *∞* if *δ*≠1.

In a sample, where *t*_1_<⋯<*t*_*J*_ denote the observed event times of the *J* subjects who experience the event of interest, the subdistribution can be estimated by the Aalen–Johansen estimator [8]:

$$\hat{F}_{\text{AJ}}(t) = \sum\limits_{j: t_{j} \leq t} \left(1 - \hat{S}(t_{j}^{-})\right) \frac{d(t_{j})}{r(t_{j}^{-})}, $$

where $\hat {S}$ is the Kaplan–Meier estimate of the all-cause survival function *S*(*t*)=Pr(*T*>*t*), *d*(*t*_*j*_) is the number of events of interest occurring at time *t*_*j*_ and $r(t_{j}^{-})$ is the number at risk (event-free and uncensored) just prior to *t*_*j*_.

Geskus [9] has shown that this estimator has two alternative representations, one as an inverse probability weighted (IPW) empirical distribution function, and one as a product-limit (PL) estimator: $\hat {F}_{\text {AJ}} = \hat {F}_{\text {IPW}} = \hat {F}_{\text {PL}}$. The representation of $\hat {F}$ as a product-limit estimator has the form:

1 $$  \hat{F}_{\text{PL}}(t) = 1 - \prod\limits_{j: t_{j} \leq t} \left(1 - \frac{d(t_{j})}{r^{*}(t_{j}^{-})} \right),  $$

where $r^{*}(t_{j}^{-})$ is a modified number at risk, which includes weighted contributions from individuals who experienced the competing event prior to time *t*_*j*_.

Specifically, if we consider a sample of *n* independent subjects with event times *T*_1_,…,*T*_*n*_ and censoring times *C*_1_,…,*C*_*n*_, where we observe *Z*_*i*_= min(*T*_*i*_,*C*_*i*_) and *δ*_*i*_, which is the type of event, or 0 if the subject was censored, then

$$r^{*}(t) = \sum\limits_{i=1}^{n} w_{i}(t), $$

where the time-dependent weight for each individual is defined as

2 $$  w_{i}(t) = \left\{ \begin{array}{ll} 1(Z_{i} \geq t) & \text{if}~\delta_{i} \in \{0, 1\} \\ \frac{G_{i}(t^{-})}{G_{i}(\min(Z_{i}^{-}, t^{-}))} & \text{if}~\delta_{i} = 2, \end{array} \right.  $$

and $G_{i}(t) = \text {Pr}(C_{i} > t\;\mid \boldsymbol {x}^{*}_{i})$ is the censoring survival distribution, which may depend on covariate vector $\boldsymbol {x}^{*}_{i}$.

For individuals who experience the event of interest or are censored, this weight is simply an indicator function representing their inclusion in the risk set over time. Individuals who experience a competing event also contribute with full weight up until their event (as the numerator and denominator are equal when *t*≤*Z*_*i*_). Afterwards, however, they continue to contribute to the modified number at risk, weighted by their conditional probability of remaining in the risk set (that is, avoiding censoring) at future event times.

If the only reason for censoring is a fixed end-of-study time point, then the potential censoring time, and hence censoring distribution, is known for every individual. In such a ‘censoring-complete’ case, each individual’s weight will be either zero or one, based on the known *C*_*i*_: *w*_*i*_(*t*)=1(*C*_*i*_>*t*), so that individuals who did not experience the event of interest are removed from the risk set at their censoring time. In the far more common scenario in which the censoring times are unknown, the censoring distribution used for the weights must be based on an estimate. The equivalence $\hat {F}_{\text {AJ}} = \hat {F}_{\text {PL}}$ holds if we use the overall Kaplan–Meier estimate of censoring survival in place of *G*_*i*_. In this paper we investigate the impact on the subdistribution estimate of using different estimates of *G*_*i*_ in calculating the weights.

### Proportional subdistribution hazards modelling

The hazard of the subdistribution takes the usual form of a hazard function, based on the improper *τ*:

$$\begin{array}{*{20}l} \gamma(t) & = {\lim}_{\Delta \rightarrow 0} \frac{1}{\Delta}~\text{Pr}(t < \tau \leq t + \Delta \mid \tau > t) \\ & = {\lim}_{\Delta \rightarrow 0} \frac{1}{\Delta}~\text{Pr}(t < T \leq t + \Delta, \delta = 1 \mid \\ & \qquad \qquad \qquad T > t~\text{or}~[\!T \leq t, \delta \neq 1]). \end{array} $$

This differs from the cause-specific hazard in that the conditional part not only includes the possibility of being event-free at time *t*, but also the possibility that a competing event has occurred prior to *t*. Its major advantage in a competing risks setting is that, unlike the cause-specific hazard, it has a one-to-one correspondence to the cumulative incidence of the event of interest.

Analogous to the cause-specific proportional hazards, or Cox, regression model [10], Fine and Gray [11] define a proportional subdistribution hazards model, where *γ*(*t*;***x***)=*γ*_0_(*t*) exp(***xβ***) for some common baseline subdistribution hazard function *γ*_0_(*t*). Under this model, exp(*β*_*p*_) represents the time-invariant subdistribution hazard ratio (SHR) associated with a one-unit increase in the *p*th component of the covariate vector ***x***, keeping everything else constant. Maximum partial likelihood estimates of ***β*** can be obtained by solving score equations, and the the null hypothesis *β*_*p*_=0 can be assessed using the associated score test or a Wald test.

For a proportional subdistribution hazards model with a single covariate *x*, the score statistic has the form

3 $$  U(\beta) = \sum\limits_{j=1}^{J} \left(x'_{j} - \frac{\sum_{i=1}^{n} w_{i}(t_{j}) x_{i} \exp(x_{i} \beta)}{\sum_{i=1}^{n} w_{i}(t_{j}) \exp(x_{i} \beta)} \right),  $$

where $x^{\prime }_{j}$ is the covariate value for the individual with an event of interest at *t*_*j*_, and *w*_*i*_(*t*) is the same time-dependent weight function shown in (2). If there are no competing events, or if competing events are treated as censored observations, *U*(*β*) is simply the score statistic for a cause-specific proportional hazards model.

This formulation makes it clear that the censoring distribution plays an important role in proportional subdistribution hazards modelling. Fine and Gray [11] give no prescription for how the censoring distribution should be estimated, using a simple Kaplan–Meier estimate over the entire sample in their example. In the case of a single binary covariate, if the censoring distribution is estimated using the Kaplan–Meier method separately in each group defined by the covariate, the score test from a Fine and Gray model is identical to Gray’s non-parametric test for comparing subdistribution hazards [12]. The optimal method for calculating censoring weights is an open question ([13], Section 5.3.1).

If it is known that censoring is covariate-dependent, it seems natural that this information should be used when estimating the censoring distribution for calculating the weights in the score statistic for the Fine and Gray model. Any implementation of a method that allows time-dependent weights to be attached to observations in a proportional hazards model can be used ([13], Section 5.7.2), but some software includes specialised functions that will calculate the weights and fit the proportional subdistribution hazards model at once. The finegray function in the survival package in R produces the weighted start-stop dataset that can be used directly in the coxph function to estimate a proportional subdistribution hazards model [14]. Alternatively, in the cmprsk package, the cengroup argument to the crr function allows the user to specify discrete groups of individuals in which separate Kaplan–Meier estimates are used to estimate the censoring distribution [15].

In the next section, we illustrate the potential efficiency losses that can occur if such information is not used, but also if the censoring distribution is misspecified due to the inclusion of unassociated factors in its estimation. Binder et al. [16] have investigated a similar issue in the context of calculating pseudo-observations when there is bias in the Aalen–Johansen estimator due to covariate-dependent censoring. However, in illustrating methods that correct for this bias, they relied on knowledge of the true model for the censoring distribution, and did not consider the case in which it is unknown.

## Methods

### Simulation study 1

Through simulations, we considered the situation in which the censoring distribution is misspecified when calculating the risk set weights for a proportional subdistribution hazards model. The simulated datasets were based on a hypothetical observational study of 300 subjects comparing two exposures labelled A and B. Reflecting the population susceptible to the event of interest, around two-thirds of subjects in the study were ‘young’ and the remaining one-third were ‘old’. Young subjects were equally likely to have either exposure A or B, while older subjects were three times more likely to have exposure B, relative to exposure A.

Events and their times were simulated under a proportional subdistribution hazards model using the algorithm described in Additional file 1, available online. The proportion of subjects who would experience the competing event was varied from 0 to 30%, as was the effect of exposure B on the subdistribution hazard of interest, from no effect to a SHR of 2.7. There was no independent effect of age on the subdistribution hazard of the event of interest.

Two types of censoring were also present in the simulations. The first was designed to simulate censoring due to a fixed end-of-study time point, with censoring times drawn from a uniform distribution to reflect constant accrual to the study. On average, approximately 10% of subjects in our sample would be censored in this way. The second type of censoring was loss to follow-up, the hazard of which was influenced by the age group of the subject. These censoring times were drawn from an exponential distribution, with the rate parameter chosen such that the proportion of young patients censored in this way was approximately 10%. The risk of loss to follow-up for older subjects was increased across our scenarios, from a hazard ratio of 1 (no difference) to 2.7 times that of younger subjects.

If either censoring time was prior to the event time for a subject, they would be censored. It is important to note that, because censoring times were simulated independently of event times, they were non-informative conditional on age group, but because exposure is associated with age, censoring time is correlated with exposure. Also under this model, censored subjects would still go on to experience an event had they been followed longer, so censoring should not be considered to be an additional type of competing event.

For each scenario we simulated 1000 datasets in R [17]. The full set of simulated data is available online (see “Availability of data and materials”). We fit four proportional subdistribution hazard models to each dataset, with exposure as the only covariate. The models differed only in how the censoring distribution was estimated in calculating the risk set weights. Model 1 used a simple pooled estimate over all subjects: $\hat {G}_{i}(t) = \hat {G}(t)$; model 2 used separate estimates in each age group: $\hat {G}_{i}(t) = \hat {G}(t \mid \text {age}_{i})$; model 3 used separate estimates for each exposure group: $\hat {G}_{i}(t) = \hat {G}(t \mid \text {exposure}_{i})$; and model 4 used a separate censoring distribution estimate for each of the four exposure–age combinations: $\hat {G}_{i}(t) = \hat {G}(t \mid \text {age}_{i},\; \text {exposure}_{i})$.

### Simulation study 2

As discussed in the introduction section, estimation in the proportional subdistribution hazards model and estimation of the subdistribution function both depend on a weight function of the form (2), which requires an estimate of the probability *G*_*i*_ that an individual remains uncensored over time. Our first simulation study was designed to examine the impact of our choice of estimator for *G*_*i*_ on the subdistribution hazard ratio estimate. In order to further investigate this issue, we undertook a second simulation study, focused on estimation of the subdistribution.

We used the same simulated data from the first study, described in the previous section and in Additional file 1, available online. For each dataset, we estimated the subdistribution in each exposure group using the product-limit form of the estimator (1), but with four different specifications of the modified number at risk *r* ^∗^. As before, these were based on estimating the censoring survival distribution separately for different groupings of exposure and age, denoted models 1–4.

The models were fit with a modified version of the cuminc function from the cmprsk package in R [15], which usually implements the Aalen–Johansen form of the estimator. The usual survfit function [14] could also be used to calculate the product–limit estimate of an appropriately weighted dataset, which could be created using crprep from the mstate package [18, 19]. Note that model 3 produces the Aalen–Johansen estimator, since the estimates and the censoring distribution are calculated separately for each exposure group. We calculated the pointwise empirical (absolute) bias and standard deviation of the estimates at 2000 time points, and took the average of these across time to obtain a summary statistic for each exposure group in each scenario.

### Multiple myeloma example

As an example of competing events in real data, we considered a cohort of 35 patients being treated for multiple myeloma at the Clinic for Stem Cell Transplantation, University Hospital Hamburg-Effendorf, Hamburg, Germany. The outcome of interest in this study was relapse of multiple myeloma, with transplant-related mortality acting as a competing risk. Of interest was a comparison of donor killer immunoglobulin-like receptor (KIR) haplotype AA versus haplotypes AB and BB together, to determine if donors with group B KIR haplotypes are associated with an improvement in time to relapse, measured as the time from transplantation [20].

**Fig. 1:**
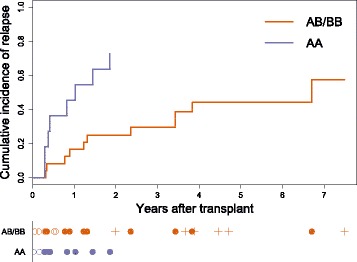
Multiple myeloma example: cumulative incidence estimates. Cumulative incidence plots for relapse in the multiple myeloma example, separated by donor KIR haplotype. The event and censoring times are shown below the plot: filled circles denote relapse, empty circles denote transplant-related death (the competing event), and plus signs are censored observations

The raw data are provided in Additional file 2 for this paper. The cohort comprised 11 patients receiving transplants from donors with type AA haplotypes and 24 receiving transplants from donors with type AB or BB. The pattern of events and censoring is illustrated in the lower panel of Fig. 1, notably showing that all of the censored observations are from patients in the AB/BB group, and all occurred after the last event in the AA group.

We fit proportional subdistribution hazards models to this data using the cmprsk package in R [15], in order to estimate the SHR associated with haplotype group. Without any additional recorded patient characteristics, we considered two options for estimating the censoring distribution to be used in calculating the risk set weights: pooled over all individuals, and separately in each haplotype group, corresponding to models 1 and 3 from our simulation study.

## Results

### Simulation study 1

**Fig. 2:**
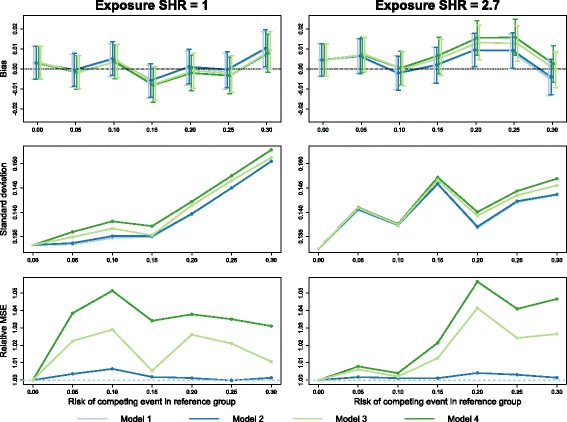
Simulations: no censoring difference. Empirical bias (*top*; with 95% confidence interval), standard deviation (*middle*) and relative mean-squared error (*bottom*) of the estimated subdistribution hazard ratio (SHR) from Fine–Gray models with four different censoring estimates: pooled (model 1), separated by age (model 2), separated by treatment (model 3) and separated by age and treatment (model 4). Simulated loss to follow-up times were drawn from an exponential distribution such that all subjects had a 10% risk of being censored in this way. The true exposure effect was zero in the left-hand column and a SHR of 2.7 in the right-hand column

The full results from the simulation study are presented in Additional file 3, available online. Figure 2 shows the empirical bias, standard deviation and relative mean-squared error (MSE) versus model 1 of the (log-)subdistribution hazard ratio estimator for two scenarios in which there was no difference in loss to follow-up between the age groups.

With no competing events, all four models produce identical estimates, as expected, with differences between the models appearing as the rate of competing events increases. Bias in the treatment effect estimate in both cases was minimal, not exceeding 0.015 on the log scale for any of the four models. Differences in bias between the models were more apparent with a large treatment effect and high risk of competing events: models 1 and 2 maintained an absolute bias of less than 0.01, while models 3 and 4 produced estimates approximately 0.005 units higher.

Models 1 and 2 were also similar in terms of variance of the effect estimate and hence mean-squared error (MSE): the difference did not exceed 0.6% for any scenario. In all cases, model 3 had a smaller MSE than model 4, with the largest differences in efficiency occurring as the risk of competing events increased. In the most extreme scenario we examined (exposure SHR = 2.7 with 30% competing events), model 3 had an MSE approximately 2.6% higher than model 1, while model 4 was approximately 4.7% higher than model 1.

**Fig. 3:**
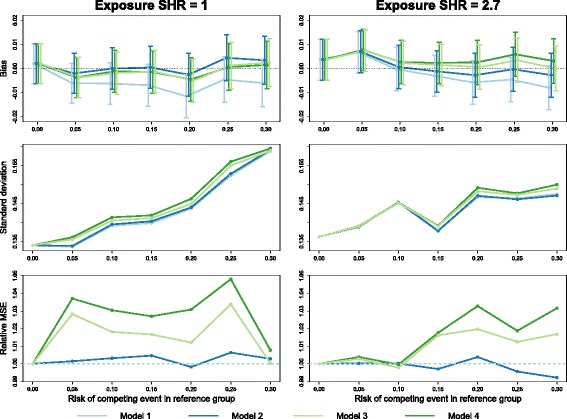
Simulations: differential censoring by age. Empirical bias (*top*; with 95% confidence interval), standard deviation (*middle*) and relative mean-squared error (*bottom*) of the estimate subdistribution hazard ratio (SHR) from Fine–Gray models with four different censoring estimates: pooled (model 1), separated by age (model 2), separated by treatment (model 3) and separated by age and treatment (model 4). Simulated data had a 10% risk of censoring due to loss to follow-up in young patients, and a censoring hazard ratio of 2.7 for older patients. The true exposure effect was zero in the left-hand column and a SHR of 2.7 in the right-hand column

Figure 3 shows a similar set of results for a loss to follow-up hazard ratio of 2.7. When there was a difference in the hazard of censoring by age, model 1 tended to underestimate the exposure effect, with a small negative bias around -0.005. Overall, models 3 and 4 also had some apparent bias, although this did not exceed 0.007 in either direction. Across all scenarios, model 2 tended to have the least bias. In terms of mean-squared error, the efficiency of models 1 and 2 were again quite similar, although with a large exposure effect and a high proportion of competing events occurring, model 2 displayed a very small efficiency gain (less than 1%) over model 1. As in Fig. 2, models 3 and 4 had poorer efficiency than models 1 and 2: in one case model 3 had 3.4% higher MSE and model 4 had 4.8% higher MSE compared to model 1.

### Simulation study 2

**Fig. 4:**
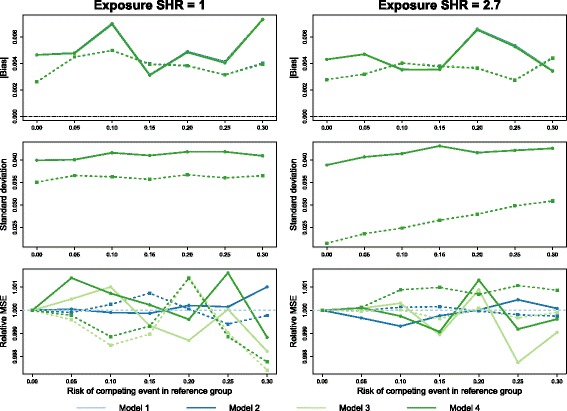
Simulations 2: no censoring difference. Empirical absolute bias (*top*), standard deviation (*middle*) and relative mean-squared error (*bottom*) of the product-limit form of the subdistribution estimator in each exposure group (A: *solid* / B: *dotted*) with weights calculated using four different censoring estimates: pooled (model 1), separated by age (model 2), separated by treatment (model 3) and separated by age and treatment (model 4). Simulated loss to follow-up times were drawn from an exponential distribution such that all subjects had a 10% risk of being censored in this way. The true exposure effect was zero in the left-hand column and a SHR of 2.7 in the right-hand column

Figure 4 shows the average of the pointwise absolute bias, standard error and relative MSE of the subdistribution estimate in each exposure group for two scenarios in which there was no difference in loss to follow-up between the age groups. In both scenarios, the bias and standard deviation differ between exposure groups, but the different models are indistinguishable from one another: the curves are completely overlapping. The minor differences between models become more apparent when we consider the MSE, but do not exceed a relative difference of 0.4%.

**Fig. 5:**
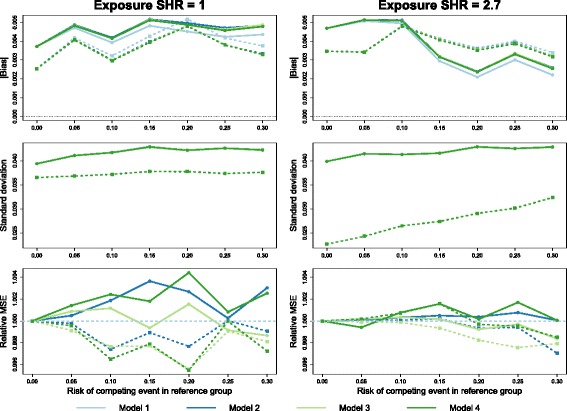
Simulations 2: differential censoring by age. Empirical absolute bias (*top*), standard deviation (*middle*) and relative mean-squared error (*bottom*) of the product-limit form of the subdistribution estimator in each exposure group (A: *solid* / B: *dotted*) with weights calculated using four different censoring estimates: pooled (model 1), separated by age (model 2), separated by treatment (model 3) and separated by age and treatment (model 4). Simulated loss to follow-up times were drawn from an exponential distribution such that all subjects had a 10% risk of being censored in this way. The true exposure effect was zero in the left-hand column and a SHR of 2.7 in the right-hand column

Figure 5 shows the same set of results for a loss to follow-up hazard ratio of 2.7. Small differences between the models in the bias are more apparent in this case, particularly model 1 versus the others. However, the standard deviations are again almost identical. The differences in MSE are larger than in the previous case, but still do not exceed a relative difference of 0.9%.

These results were representative of the full set of scenarios that we investigated: the differences in bias and variance between models being much smaller than any differences in these measures between exposure groups.

### Multiple myeloma example

**Fig. 6:**
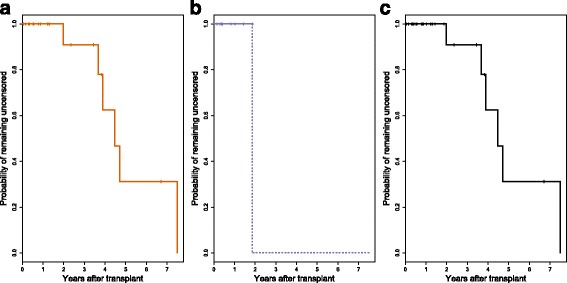
Multiple myeloma example: censoring distribution estimates. Estimated censoring distributions for the multiple myeloma example: **a** in the AB/BB group alone; **b** in the AA group alone (with extrapolation); **c** pooled over all individuals. Tick marks denote the times at which events occurred

The main panel of Fig. 1 shows the estimated subdistribution of relapse in each group. The Kaplan–Meier estimates of the censoring distribution that are used in the calculation of the proportional subdistribution hazards model are shown in Fig. 6: separately in each haplotype group (A, B) and pooled over all subjects (C). Note that the pooled curve (C) is identical to the curve for the AB/BB group, because only AB/BB patients remain in the censoring risk set at the time of the first censoring event.

Using the pooled censoring distribution, the estimated SHR for AA haplotypes compared to AB/BB is 2.56, with 95% confidence interval 1.00–6.55 and associated *p*-value 0.051. Using separate censoring distribution estimates, we obtain a SHR of 4.10 and 95% confidence interval 1.49–11.29. The associated *p*-value in this case is 0.0063.

Because the estimated censoring distribution is the same whether calculated over all patients or in the AB/BB group alone, the difference in the parameter estimates is entirely driven by the three patients in the AA group who experienced the competing event. In the analysis that uses separate censoring distribution estimates, the AA censoring distribution cannot be estimated beyond two years, and the censoring survival estimate is set to zero after that time (as shown in Fig. 6 b). This means that these three patients are removed from the risk set in the calculation of the score function at the four event times after two years.

## Discussion

### Simulation study 1

With no difference in the true censoring distribution between groups, models 1 and 2 showed very similar performance; although model 1 appeared to have a slight advantage when the true effect of the exposure was large, this could be ascribed to random variation. The largest difference was in the performance of models 3 and 4, particularly in the variance of the effect estimate, showing noticeably lower efficiency than models 1 and 2. This suggests that when there is no true difference in the hazard of being censored, the best risk set weights will, unsurprisingly, result from a pooled censoring distribution estimate.

When there was a difference in the hazard of censoring by age group, model 1—which does not take this into account—appeared to have some bias. Although the lower variance of its estimator meant that it generally had the best efficiency in terms of MSE, model 2 outperformed it in some scenarios as the risk of competing risks increased and estimation of the risk set weights would have a greater impact. Model 3 again had better performance than model 4, most likely because of the lower variability in its estimates of the censoring distributions.

The reason for the apparently superior performance of model 2 compared to model 3 in both scenarios is unclear, but may suggest that the form of the score statistic (3) is not correct when the time-to-censoring distribution depends on covariates ([13], Section 5.3.1). Nonetheless, our results suggest that better performance of this SHR estimator may be attained if the risk set weights are not made to depend on the covariate of interest, if possible.

### Simulation study 2

The results from our second simulation study do not seem to offer a clear explanation for the phenomena we observed in the estimation of the subdistribution hazard ratio. When there was no difference in censoring between age groups, the random differences between the four models did not become more apparent as the rate of competing events increased, and the models were almost identical across all scenarios.

When the hazard of censoring differed between age groups, differences in between the models—particularly model 1 versus the non-pooled versions—were larger, but the relative MSEs were substantially smaller than we observed in the first simulations, and did not point to a clearly superior approach.

The most apparent pattern from these simulations was the difference in standard deviation between the exposure groups, with exposure B having smaller variance. This was expected under the setup of the simulations, because the sample size of individuals with exposure B was consistently larger than that of exposure A.

### Multiple myeloma example

The large discrepancy between the estimates from either model is unsurprising, considering the potential differences seen in our simulations and the contrast between the estimated (extrapolated) censoring distributions for each group in this example. But without background information, drawing a conclusion from this analysis is troublesome: in one case, the 95% confidence interval suggests that the null is a plausible value for the SHR, whereas in the other, the lower confidence limit is well above unity and the *p*-value suggests that we have strong evidence against a null hypothesis of no difference.

In this example, the only cause of censoring was end-of-study, and there is no reason to suspect that the hazard of this should differ between the two groups. Had the three AA patients who experienced the competing event remained alive, we expect that they would have been censored in a similar fashion to those in the AB/BB group. This suggests that using the pooled censoring distribution to calculate weights is the more logical option, and we expect that the SHR estimate based on this will be more reliable.

This is supported by the simulation results, where in both scenarios, models 3 and 4 showed poorer performance than models 1 and 2. That is, when the risk set weights were allowed to differ by the covariate of interest, the resulting estimates showed poorer efficiency than those based on a pooled estimate.

## Conclusions

In this paper, we have highlighted the crucial role that censoring plays in a common method for regression analysis of survival data in the presence of competing risks. The Fine and Gray proportional subdistribution hazards model uses an estimate of the censoring distribution in calculating the weighted contribution to the risk set made by individuals that experience the competing event.

Using simulations, we examined the importance of correctly identifying the covariates that affect the censoring distribution. Calculating risk set weights separately when a pooled censoring distribution was more appropriate led to some loss of efficiency. When censoring was covariate-dependent, possible bias in the SHR estimate could be reduced by ensuring that censoring weights were correctly specified, but if the covariate of interest was used in this calculation, the resulting estimates had higher variance. This was apparent even when the rate of competing events was as low as 5%.

However, our second set of simulations, examining different weighting options in the product-limit form of the subdistribution estimator, did not display similarly large differences. This suggests that future work could focus on examining why the choice of weights appears to disproportionately affect estimation of the SHR compared to the subdistribution itself.

Although our examples were quite simple, using only binary covariates, we expect the same idea to hold true for more complex scenarios: more accurate estimation of the censoring distribution will improve estimation of the subdistribution hazard ratio. However, if a more complicated model for censoring is estimated in calculating risk set weights, the asymptotic properties of the SHR estimator may be difficult to identify. The approach presented by Ruan and Gray [21] uses multiple imputation to simulate censoring-complete data, which can then be used in a standard analysis method, but this still presents the problem of identifying the group of covariates that should be included in the imputation model.

It is important to consider the assumptions underlying these methods, and determine if their application is appropriate in each situation. In the multiple myeloma example, even though the pattern of censoring in the two groups appeared to be very different, this was a result of the chance occurrence that all of the patients in one group were observed to experience an event. Where it is not clear whether some types of censoring are perhaps informative and should be treated as a competing event, Siannis et al. [22] have presented a method for a sensitivity analysis that can help assess the possible impact of an incorrect assumption.

Estimation of the proportional subdistribution hazards model has been adapted to allow for both left truncation and right censoring [9]. This approach also includes weights that depend on an estimate of the entry time distribution, for which similar issues would be relevant. An estimate of the censoring distribution is also used in competing risk analysis methods that are based on the conditional (rather than marginal) probability of an event. For example, Pepe and Mori [23] and Lunn [24] include a weight function in their test statistics, but do not prescribe its specific form. In their respective examples, Pepe and Mori [23] use separate censoring distribution estimates in the weight function, while Lunn [24] uses the pooled estimate of the overall censoring survival function. Beyond its use in specific models for competing events, the concept of inverse probability of censoring weights is widely employed to attempt to account for potentially informative censoring or non-compliance in medical studies (e.g. [4, 25, 26]). While we expect that considerations similar to those discussed here will also apply to these other approaches, detailed investigations that focus on specific models would provide useful information.

We hope that this paper encourages increased consideration of the potentially large impact of censoring on competing risks methods when planning, performing and reporting such analyses.

## Additional files

Additional file 1 Simulating proportional subdistribution hazards. Describes an algorithm for simulating survival data with competing risks under a proportional subdistribution hazards model with censoring, as used in the simulation study in this paper. (PDF 157 kb)

Additional file 2 Multiple myeloma data. Survival data from the multiple myeloma example used in this paper, including the donor KIR haplotype, event times and types (0 = censored, 1 = relapse, 2 = transplant-related death [competing event]) for 35 patients. (CSV 581 bytes)

Additional file 3 Simulation results. Tables containing the complete results from the simulation study described in this paper. (PDF 92.7 kb)

## Abbreviations

AJ: Aalen–Johansen
IPW: Inverse-probability weighting
KIR: Killer immunoglobulin-like receptor
PL: Product-limit
SHR: Subdistribution hazard ratio
