# Supplementary material for: The importance of censoring in competing risks analysis of the subdistribution hazard
Source: BMC Med Res Methodol. 2017 Apr 4;17:52. doi: 10.1186/s12874-017-0327-3 (PMC5379776; doi:10.1186/s12874-017-0327-3)
Supplement: Supplementary file 1 — Simulating proportional subdistribution hazards. Describes an algorithm for simulating survival data with competing risks under a proportional subdistribution hazards model with censoring, as used in the simulation study in this paper. (PDF 157 kb) [file 12874_2017_327_MOESM1_ESM.pdf]

Additional file 1 for "The importance of censoring in competing risks analysis of the subdistribution hazard"

Mark W Donoghoe and Val Gebski

Simulating competing risks data under the proportional subdistribution hazards model

Let T_i denote the time of the first event for subject i, and δ_i the type of that first event (where $\delta_i = 1$ denotes the event of interest and $\delta_i = 2$ any competing risk, without loss of generality). Let $p(\boldsymbol{x}_i) = \Pr(T_i < \infty, \delta_i = 1 \mid \boldsymbol{x}_i)$ be the probability of experiencing the event of interest for a subject with covariate vector $\boldsymbol{x}_i$, and $p_0 = p(\boldsymbol{0})$ is this probability for an individual with the 'reference' covariate vector. $p(\boldsymbol{x}_i) = 1$ for all $\boldsymbol{x}_i$ implies that the event of interest will eventually occur for every subject. $q(\boldsymbol{x}_i) = 1 - p(\boldsymbol{x}_i) = \Pr(T_i < \infty, \delta_i = 2 \mid \boldsymbol{x}_i)$ is the probability of experiencing the competing risk, and $q_0 = 1 - p_0$.

In a proportional subdistribution hazards model with parameter vector $\boldsymbol{\beta}$, $\exp(\beta_p)$ is the subdistribution hazard ratio associated with a one-unit increase in the pth component of the covariate vector.

A proportional subdistribution hazards model can be obtained by defining the subdistribution for the event of interest as

$$\begin{aligned} F(t; \boldsymbol{x}_i) &= \Pr(T_i \le t, \delta_i = 1 \mid \boldsymbol{x}_i) \\ &= 1 - \left[1 - p_0 \left\{1 - \exp(-t)\right\}\right]^{\exp(\boldsymbol{\beta}\boldsymbol{x}_i)} . \end{aligned}$$

That we have the desired model can be verified by using the relation

$$F(t; \boldsymbol{x}_i) = 1 - \exp\left\{ - \int_0^t \gamma(s; \boldsymbol{x}_i)\,\mathrm{d}s \right\}$$

to obtain

$$\begin{aligned} \gamma(t; \boldsymbol{x}_i) &= \frac{p_0 \exp(-t)}{1 - p_0(1 - \exp(-t))} \exp(\boldsymbol{\beta}\boldsymbol{x}_i) \\ &= \gamma_0(t) \exp(\boldsymbol{\beta}\boldsymbol{x}_i) \end{aligned}$$

To simulate the competing risks data, we first generate the event type δ_i for each subject using a binomial random variable with the probability of the event of interest occurring as the first event:

$$\Pr(\delta_i = 1 \mid \boldsymbol{x}_i) = 1 - (1 - p_0)^{\exp(\boldsymbol{\beta}\boldsymbol{x}_i)}$$

If the simulated $\delta_i = 1$, the distribution of event times is

$$\begin{aligned}\Pr(T_i \le t \mid \delta_i = 1, \boldsymbol{x}_i) &= \frac{\Pr(T_i \le t, \delta_i = 1 \mid \boldsymbol{x}_i)}{\Pr(\delta_i = 1 \mid \boldsymbol{x}_i)} \\ &= \frac{1 - [1 - p_0\{1 - \exp(-t)\}]^{\exp(\boldsymbol{\beta}\boldsymbol{x}_i)}}{1 - (1 - p_0)^{\exp(\boldsymbol{\beta}\boldsymbol{x}_i)}},\end{aligned}$$

and we can use the inverse of this cumulative distribution function to simulate event times T_i from simulated uniform random variables $U_i \sim U[0,1]$ as

$$T_i = -\log\left(\frac{\left(1 - U_i\left[1 - (1 - p_0)^{\exp(\boldsymbol{\beta}\boldsymbol{x}_i)}\right]\right)^{\exp(-\boldsymbol{\beta}\boldsymbol{x}_i)} - (1 - p_0)}{p_0}\right)$$

If $\delta_i = 2$, we can assign event times in any way we see fit, possibly including a relationship with some covariate vector $\boldsymbol{z}_i$ (which may include components of $\boldsymbol{x}_i$). In our simulation study, we simply used exponentially distributed event times for all individuals:

$$\Pr(T_i \le t \mid \delta_i = 2) = 1 - \exp(-t)$$

Censoring times C_i are simulated independently of event times, but may be related to the covariates $\boldsymbol{x}_i$ and/or $\boldsymbol{z}_i$. If end of follow-up is the only reason for censoring, C_i may be fixed at a certain time, or generated from a uniform distribution in the case of a clinical trial with a constant accrual rate.

For our simulations, we generated two potential censoring times for each individual: C_{i1} and C_{i2}. C_{i1} represented censoring due to end of study with constant accrual, and was drawn from a uniform distribution $U[0, c_{\mathrm{m}}]$, with c_{m} chosen so that the desired proportion of subjects p_{C_1} would have a censoring time prior to their event time T_i, using:

$$p_{\mathrm{C}_1} \approx \Pr(C_{i1} < T_i \mid x_i = 0) = \frac{1}{c_{\mathrm{m}}} - e^{-c_{\mathrm{m}}}.$$

In our simulations, we used $p_{\mathrm{C}_1} = 0.1$, so $c_{\mathrm{m}} \approx 10$.

C_{i2} represented censoring due to loss to follow-up, drawn independently from an exponential distribution, depending on some covariate vector $\boldsymbol{\eta}_i$ (in our case, age group):

$$\Pr(C_{i2} \le c \mid \boldsymbol{\eta}_i) = 1 - \exp(-c\lambda_{\mathrm{C}} \exp(\boldsymbol{\beta}_{\mathrm{C}}\boldsymbol{\eta}_i))$$

with λ_{C} chosen so that approximately $p_{\mathrm{C}_2} = 10\%$ of subjects with $\eta_i = 0$ (young) would have $C_{i2} < T_i$:

$$p_{\mathrm{C}_2} \approx \Pr(C_{i2} < T_i \mid x_i = 0, \eta_i = 0) = \frac{\lambda_{\mathrm{C}}}{\lambda_{\mathrm{C}} + 1},$$

that is $\lambda_{\mathrm{C}} = 1/9$, and β_{C} varied across scenarios. Each individual's censoring time was taken as the first of either type, that is, $C_i = \min(C_{i1}, C_{i2})$.

Each subject's time-to-event Z_i is then the minimum of T_i and C_i, and we set $\delta_i = 0$ if the subject is censored before experiencing an event (that is, if $C_i < T_i$).
