# Supplementary material for: The importance of censoring in competing risks analysis of the subdistribution hazard
Source: BMC Med Res Methodol. 2017 Apr 4;17:52. doi: 10.1186/s12874-017-0327-3 (PMC5379776; doi:10.1186/s12874-017-0327-3)
Supplement: Supplementary file 3 — Simulation results. Tables containing the complete results from the simulation study described in this paper. (PDF 92.7 kb) [file 12874_2017_327_MOESM3_ESM.pdf]

Additional file 3 for
"The importance of censoring in competing risks analysis of the subdistribution hazard"

Mark W Donoghoe and Val Gebski

Results from simulation study

The following tables present the full results from the simulation study described in the main paper. The algorithm used to generate the simulated datasets is described in Additional file 1. In each case, the approximate rate of censoring due to end-of-study was fixed at 10% and the approximate rate of young subjects who were censored due to loss to follow-up was 10%.

Each table shows a different hazard ratio for censoring due to loss to follow-up between old and young subjects, varying between 1.00 and 2.72. Within each table, the true subdistribution hazard ratio for exposure was varied between 1.00 and 2.72, and the risk of the competing event in the reference exposure group (q_0) was varied between 0% and 30%.

The empirical bias is the observed average bias across the 1000 simulations, and the SD is the sample standard deviation of the 1000 estimates. The standard error of the estimated bias is $\text{SD}/\sqrt{1000}$. MSE was calculated as $\text{Bias}^2 + \text{SD}^2$, and is presented relative to Model 1. Note that when $q_0 = 0$, so there are no competing events, all four models are identical.

Censoring HR = 1.00 (no difference)										
Exposure SHR		1.00			1.65			2.72		
q_0	Model	Bias	SD	Rel MSE	Bias	SD	Rel MSE	Bias	SD	Rel MSE
0.00	Model 1	0.003	0.133	1.000	0.002	0.130	1.000	0.004	0.133	1.000
	Model 2	0.003	0.133	1.000	0.002	0.130	1.000	0.004	0.133	1.000
	Model 3	0.003	0.133	1.000	0.002	0.130	1.000	0.004	0.133	1.000
	Model 4	0.003	0.133	1.000	0.002	0.130	1.000	0.004	0.133	1.000
0.05	Model 1	-0.001	0.133	1.000	0.005	0.127	1.000	0.006	0.140	1.000
	Model 2	-0.001	0.134	1.004	0.005	0.127	1.000	0.006	0.141	1.002
	Model 3	-0.002	0.135	1.022	0.007	0.126	1.000	0.007	0.141	1.006
	Model 4	-0.002	0.136	1.038	0.008	0.127	1.002	0.007	0.141	1.008
0.10	Model 1	0.004	0.135	1.000	0.009	0.132	1.000	-0.002	0.137	1.000
	Model 2	0.005	0.135	1.006	0.009	0.132	0.996	-0.002	0.137	1.001
	Model 3	0.004	0.137	1.029	0.011	0.134	1.022	-0.000	0.137	1.002
	Model 4	0.003	0.138	1.051	0.012	0.134	1.030	0.000	0.138	1.004
0.15	Model 1	-0.006	0.135	1.000	-0.001	0.132	1.000	0.002	0.146	1.000
	Model 2	-0.006	0.135	1.002	-0.001	0.132	1.002	0.002	0.146	1.001
	Model 3	-0.007	0.135	1.005	-0.001	0.134	1.031	0.006	0.147	1.013
	Model 4	-0.008	0.137	1.034	0.000	0.135	1.053	0.007	0.147	1.021
0.20	Model 1	0.000	0.140	1.000	0.003	0.138	1.000	0.009	0.137	1.000
	Model 2	0.001	0.140	1.001	0.004	0.138	1.004	0.009	0.137	1.004
	Model 3	-0.001	0.141	1.026	0.004	0.140	1.029	0.013	0.139	1.041
	Model 4	-0.002	0.142	1.038	0.005	0.141	1.048	0.015	0.140	1.057
0.25	Model 1	-0.001	0.145	1.000	0.003	0.144	1.000	0.009	0.142	1.000
	Model 2	-0.000	0.145	1.000	0.004	0.145	1.005	0.009	0.142	1.003
	Model 3	-0.003	0.147	1.021	0.004	0.146	1.029	0.013	0.144	1.024
	Model 4	-0.003	0.147	1.035	0.005	0.148	1.048	0.016	0.144	1.041
0.30	Model 1	0.009	0.150	1.000	0.003	0.144	1.000	-0.005	0.144	1.000
	Model 2	0.010	0.150	1.001	0.004	0.144	1.004	-0.004	0.144	1.001
	Model 3	0.009	0.151	1.011	0.004	0.145	1.026	-0.000	0.146	1.026
	Model 4	0.008	0.153	1.031	0.005	0.148	1.056	0.002	0.147	1.047

Table 1: Empirical bias, standard deviation and relative MSE of the estimators of the exposure subdistribution hazard ratio (SHR) from four different models, based on 1000 simulations for each scenario. The true exposure SHR and approximate proportion of subjects in the reference group experiencing the competing event (q_0) were varied, and the hazard of censoring from loss to follow-up between age groups was fixed at 1.00 (old versus young).

Censoring HR = 1.28										
Exposure SHR		1.00			1.65			2.72		
q_0	Model	Bias	SD	Rel MSE	Bias	SD	Rel MSE	Bias	SD	Rel MSE
0.00	Model 1	0.003	0.130	1.000	0.001	0.131	1.000	0.010	0.136	1.000
	Model 2	0.003	0.130	1.000	0.001	0.131	1.000	0.010	0.136	1.000
	Model 3	0.003	0.130	1.000	0.001	0.131	1.000	0.010	0.136	1.000
	Model 4	0.003	0.130	1.000	0.001	0.131	1.000	0.010	0.136	1.000
0.05	Model 1	0.000	0.138	1.000	0.001	0.129	1.000	0.009	0.136	1.000
	Model 2	0.001	0.138	1.002	0.002	0.129	1.002	0.009	0.136	1.000
	Model 3	-0.001	0.141	1.040	0.003	0.130	1.023	0.010	0.136	1.004
	Model 4	-0.001	0.142	1.052	0.004	0.131	1.036	0.010	0.136	1.007
0.10	Model 1	-0.002	0.130	1.000	-0.003	0.135	1.000	0.011	0.136	1.000
	Model 2	-0.001	0.131	1.002	-0.002	0.135	1.000	0.011	0.136	1.002
	Model 3	-0.002	0.133	1.038	-0.001	0.137	1.030	0.013	0.136	1.006
	Model 4	-0.002	0.135	1.064	0.001	0.137	1.043	0.013	0.136	1.007
0.15	Model 1	-0.002	0.139	1.000	-0.001	0.137	1.000	0.003	0.142	1.000
	Model 2	0.000	0.140	1.002	0.000	0.137	1.001	0.004	0.142	1.002
	Model 3	-0.002	0.139	0.997	0.001	0.138	1.014	0.007	0.143	1.013
	Model 4	-0.002	0.141	1.021	0.003	0.139	1.032	0.008	0.143	1.020
0.20	Model 1	0.003	0.142	1.000	-0.000	0.138	1.000	0.016	0.142	1.000
	Model 2	0.005	0.142	1.005	0.001	0.138	1.004	0.017	0.142	1.003
	Model 3	0.003	0.143	1.026	0.003	0.141	1.043	0.020	0.144	1.030
	Model 4	0.002	0.145	1.044	0.003	0.142	1.062	0.023	0.144	1.044
0.25	Model 1	-0.001	0.151	1.000	0.007	0.137	1.000	0.002	0.140	1.000
	Model 2	0.002	0.152	1.003	0.009	0.137	1.006	0.003	0.141	1.004
	Model 3	0.001	0.152	1.011	0.010	0.139	1.034	0.007	0.143	1.033
	Model 4	-0.001	0.154	1.032	0.011	0.140	1.057	0.011	0.144	1.053
0.30	Model 1	-0.003	0.157	1.000	-0.002	0.146	1.000	-0.002	0.146	1.000
	Model 2	-0.001	0.157	0.998	-0.000	0.146	0.997	-0.000	0.146	0.999
	Model 3	-0.002	0.157	1.006	-0.001	0.148	1.036	0.003	0.147	1.013
	Model 4	-0.002	0.158	1.016	0.000	0.150	1.054	0.006	0.148	1.029

Table 2: Empirical bias, standard deviation and relative MSE of the estimators of the exposure subdistribution hazard ratio (SHR) from four different models, based on 1000 simulations for each scenario. The true exposure SHR and approximate proportion of subjects in the reference group experiencing the competing event (q_0) were varied, and the hazard of censoring from loss to follow-up between age groups was fixed at 1.28 (old versus young).

Censoring HR = 1.65										
Exposure SHR		1.00			1.65			2.72		
q_0	Model	Bias	SD	Rel MSE	Bias	SD	Rel MSE	Bias	SD	Rel MSE
0.00	Model 1	-0.002	0.133	1.000	0.002	0.132	1.000	0.001	0.138	1.000
	Model 2	-0.002	0.133	1.000	0.002	0.132	1.000	0.001	0.138	1.000
	Model 3	-0.002	0.133	1.000	0.002	0.132	1.000	0.001	0.138	1.000
	Model 4	-0.002	0.133	1.000	0.002	0.132	1.000	0.001	0.138	1.000
0.05	Model 1	-0.003	0.136	1.000	0.006	0.133	1.000	0.007	0.139	1.000
	Model 2	-0.001	0.136	1.004	0.006	0.134	1.002	0.007	0.139	1.003
	Model 3	-0.003	0.139	1.044	0.008	0.135	1.018	0.007	0.139	1.006
	Model 4	-0.002	0.140	1.059	0.008	0.135	1.023	0.007	0.139	1.007
0.10	Model 1	-0.005	0.135	1.000	0.004	0.130	1.000	0.005	0.138	1.000
	Model 2	-0.002	0.136	1.005	0.006	0.130	1.002	0.006	0.138	1.000
	Model 3	-0.003	0.137	1.015	0.006	0.132	1.033	0.007	0.138	1.001
	Model 4	-0.003	0.138	1.031	0.008	0.133	1.047	0.008	0.138	1.005
0.15	Model 1	0.001	0.139	1.000	0.007	0.138	1.000	0.002	0.140	1.000
	Model 2	0.004	0.139	1.004	0.009	0.139	1.012	0.003	0.140	1.004
	Model 3	0.004	0.141	1.028	0.011	0.141	1.036	0.006	0.141	1.027
	Model 4	0.004	0.143	1.048	0.013	0.142	1.055	0.008	0.142	1.034
0.20	Model 1	-0.002	0.143	1.000	0.004	0.146	1.000	0.003	0.141	1.000
	Model 2	0.001	0.144	1.007	0.007	0.147	1.005	0.004	0.141	1.000
	Model 3	-0.002	0.144	1.016	0.009	0.148	1.023	0.007	0.142	1.019
	Model 4	-0.003	0.146	1.042	0.010	0.149	1.040	0.009	0.142	1.024
0.25	Model 1	-0.011	0.157	1.000	-0.002	0.139	1.000	0.003	0.143	1.000
	Model 2	-0.006	0.158	1.003	0.001	0.139	1.006	0.005	0.143	1.000
	Model 3	-0.009	0.159	1.017	0.002	0.140	1.016	0.009	0.144	1.021
	Model 4	-0.010	0.161	1.049	0.003	0.142	1.043	0.011	0.145	1.034
0.30	Model 1	-0.007	0.159	1.000	-0.003	0.149	1.000	0.006	0.148	1.000
	Model 2	-0.003	0.159	1.000	0.001	0.149	1.003	0.008	0.149	1.008
	Model 3	-0.004	0.160	1.010	0.000	0.149	1.012	0.011	0.150	1.029
	Model 4	-0.005	0.161	1.025	0.001	0.151	1.027	0.014	0.151	1.043

Table 3: Empirical bias, standard deviation and relative MSE of the estimators of the exposure subdistribution hazard ratio (SHR) from four different models, based on 1000 simulations for each scenario. The true exposure SHR and approximate proportion of subjects in the reference group experiencing the competing event (q_0) were varied, and the hazard of censoring from loss to follow-up between age groups was fixed at 1.65 (old versus young).

Censoring HR = 2.12										
Exposure SHR		1.00			1.65			2.72		
q_0	Model	Bias	SD	Rel MSE	Bias	SD	Rel MSE	Bias	SD	Rel MSE
0.00	Model 1	0.009	0.136	1.000	0.002	0.128	1.000	-0.001	0.135	1.000
	Model 2	0.009	0.136	1.000	0.002	0.128	1.000	-0.001	0.135	1.000
	Model 3	0.009	0.136	1.000	0.002	0.128	1.000	-0.001	0.135	1.000
	Model 4	0.009	0.136	1.000	0.002	0.128	1.000	-0.001	0.135	1.000
0.05	Model 1	-0.009	0.134	1.000	-0.004	0.131	1.000	0.004	0.138	1.000
	Model 2	-0.006	0.134	1.002	-0.002	0.131	1.004	0.004	0.138	1.000
	Model 3	-0.008	0.136	1.023	-0.001	0.132	1.014	0.005	0.138	1.002
	Model 4	-0.008	0.137	1.040	-0.001	0.133	1.022	0.005	0.138	1.004
0.10	Model 1	-0.005	0.139	1.000	0.001	0.136	1.000	0.004	0.135	1.000
	Model 2	-0.000	0.139	1.000	0.003	0.136	0.996	0.005	0.135	0.998
	Model 3	-0.002	0.140	1.013	0.004	0.137	1.019	0.007	0.136	1.011
	Model 4	-0.002	0.141	1.030	0.005	0.137	1.025	0.007	0.136	1.011
0.15	Model 1	-0.003	0.137	1.000	-0.006	0.135	1.000	0.005	0.142	1.000
	Model 2	0.003	0.137	1.004	-0.002	0.135	1.001	0.006	0.142	1.006
	Model 3	0.001	0.138	1.012	-0.001	0.137	1.026	0.009	0.142	1.006
	Model 4	0.001	0.139	1.028	0.001	0.138	1.046	0.011	0.143	1.014
0.20	Model 1	-0.001	0.151	1.000	-0.000	0.139	1.000	-0.012	0.142	1.000
	Model 2	0.005	0.151	1.008	0.004	0.140	1.007	-0.010	0.142	0.996
	Model 3	0.002	0.152	1.025	0.006	0.142	1.038	-0.007	0.144	1.018
	Model 4	0.002	0.154	1.040	0.007	0.143	1.056	-0.005	0.145	1.029
0.25	Model 1	-0.002	0.147	1.000	0.004	0.140	1.000	-0.002	0.142	1.000
	Model 2	0.004	0.147	1.003	0.009	0.141	1.009	0.001	0.142	0.999
	Model 3	0.003	0.148	1.021	0.010	0.141	1.020	0.005	0.143	1.011
	Model 4	0.003	0.150	1.044	0.012	0.143	1.049	0.007	0.144	1.024
0.30	Model 1	-0.001	0.158	1.000	-0.006	0.149	1.000	0.000	0.149	1.000
	Model 2	0.006	0.158	1.012	-0.001	0.150	1.002	0.004	0.149	1.003
	Model 3	0.005	0.159	1.017	-0.002	0.151	1.021	0.007	0.150	1.020
	Model 4	0.004	0.160	1.034	-0.001	0.153	1.041	0.010	0.151	1.036

Table 4: Empirical bias, standard deviation and relative MSE of the estimators of the exposure subdistribution hazard ratio (SHR) from four different models, based on 1000 simulations for each scenario. The true exposure SHR and approximate proportion of subjects in the reference group experiencing the competing event (q_0) were varied, and the hazard of censoring from loss to follow-up between age groups was fixed at 2.12 (old versus young).

Censoring HR = 2.72										
Exposure SHR		1.00			1.65			2.72		
q_0	Model	Bias	SD	Rel MSE	Bias	SD	Rel MSE	Bias	SD	Rel MSE
0.00	Model 1	0.002	0.134	1.000	0.001	0.135	1.000	0.004	0.136	1.000
	Model 2	0.002	0.134	1.000	0.001	0.135	1.000	0.004	0.136	1.000
	Model 3	0.002	0.134	1.000	0.001	0.135	1.000	0.004	0.136	1.000
	Model 4	0.002	0.134	1.000	0.001	0.135	1.000	0.004	0.136	1.000
0.05	Model 1	-0.006	0.134	1.000	-0.006	0.134	1.000	0.007	0.139	1.000
	Model 2	-0.002	0.134	1.001	-0.004	0.135	1.005	0.007	0.139	1.000
	Model 3	-0.004	0.136	1.028	-0.003	0.136	1.021	0.008	0.139	1.003
	Model 4	-0.004	0.136	1.037	-0.003	0.136	1.029	0.008	0.139	1.004
0.10	Model 1	-0.006	0.139	1.000	-0.009	0.137	1.000	-0.000	0.145	1.000
	Model 2	-0.000	0.140	1.003	-0.006	0.137	1.001	0.001	0.145	1.000
	Model 3	-0.002	0.141	1.018	-0.003	0.138	1.015	0.002	0.145	0.998
	Model 4	-0.001	0.141	1.030	-0.002	0.139	1.027	0.003	0.145	1.000
0.15	Model 1	-0.007	0.140	1.000	0.004	0.138	1.000	-0.003	0.138	1.000
	Model 2	0.000	0.140	1.005	0.010	0.138	1.014	-0.001	0.138	0.997
	Model 3	-0.001	0.141	1.017	0.011	0.140	1.038	0.001	0.139	1.016
	Model 4	-0.001	0.142	1.027	0.012	0.142	1.070	0.002	0.139	1.018
0.20	Model 1	-0.011	0.144	1.000	-0.003	0.132	1.000	-0.006	0.147	1.000
	Model 2	-0.003	0.144	0.998	0.003	0.132	1.000	-0.003	0.147	1.004
	Model 3	-0.005	0.145	1.012	0.004	0.133	1.014	0.001	0.148	1.020
	Model 4	-0.005	0.146	1.031	0.005	0.133	1.026	0.003	0.149	1.033
0.25	Model 1	-0.005	0.152	1.000	0.000	0.138	1.000	-0.005	0.146	1.000
	Model 2	0.004	0.153	1.006	0.008	0.138	1.009	-0.000	0.146	0.996
	Model 3	0.001	0.155	1.034	0.008	0.138	0.999	0.003	0.147	1.013
	Model 4	0.001	0.156	1.048	0.010	0.139	1.021	0.006	0.148	1.019
0.30	Model 1	-0.006	0.159	1.000	-0.004	0.152	1.000	-0.008	0.148	1.000
	Model 2	0.003	0.159	1.003	0.004	0.152	1.003	-0.003	0.147	0.992
	Model 3	0.002	0.159	1.000	0.003	0.152	1.003	-0.000	0.149	1.017
	Model 4	0.002	0.160	1.008	0.005	0.153	1.016	0.003	0.150	1.032

Table 5: Empirical bias, standard deviation and relative MSE of the estimators of the exposure subdistribution hazard ratio (SHR) from four different models, based on 1000 simulations for each scenario. The true exposure SHR and approximate proportion of subjects in the reference group experiencing the competing event (q_0) were varied, and the hazard of censoring from loss to follow-up between age groups was fixed at 2.72 (old versus young).
